# Indigenous Non-*Saccharomyces* Yeasts With *β*-Glucosidase Activity in Sequential Fermentation With *Saccharomyces cerevisiae*: A Strategy to Improve the Volatile Composition and Sensory Characteristics of Wines

**DOI:** 10.3389/fmicb.2022.845837

**Published:** 2022-05-12

**Authors:** Pingping Gao, Shuai Peng, Faisal Eudes Sam, Yatong Zhu, Lihong Liang, Min Li, Jing Wang

**Affiliations:** ^1^College of Food Science and Engineering, Gansu Agricultural University, Lanzhou, China; ^2^Gansu Key Lab of Viticulture and Enology, Lanzhou, China

**Keywords:** *β*-glucosidase, *Hanseniaspora uvarum*, *Meyerozyma guilliermondii*, yeast, fermentation, sequential inoculation, wine aroma

## Abstract

Non-*Saccharomyces* (NS) yeasts with high *β*-glucosidase activity play a vital role in improving the aroma complexity of wines by releasing aroma compounds from glycosidic precursors during fermentation. In this study, the effect of sequential inoculation fermentation of *Meyerozyma guilliermondii* NM218 and *Hanseniaspora uvarum* BF345 with two *Saccharomyces cerevisiae* strains [Vintage Red™ (VR) and Aroma White™ (AW)] on volatile compounds and sensory characteristics of wines was investigated. Prior to winemaking trials, the sequential inoculation times of the two NS yeasts were evaluated in synthetic must, based on changes in strain population and enzyme activity. The intervals for inoculation of NM218 and BF345 with the *S. cerevisiae* strains were 48 and 24 h, respectively. In the main experiment, sequential inoculation fermentations of the two strains with *S. cerevisiae* were carried out in Cabernet Sauvignon (CS) and Chardonnay (CH) grape must. The oenological parameters, volatile composition, and sensory characteristics of the final wines were assessed. No clear differences were observed in the oenological parameters of the sequentially fermented CH wines compared with the control, except for residual sugar and alcohol. However, in CS wines, the total acid contents were significantly lower in the wines fermented by sequential inoculation compared to the control. Both NM218 and BF345 improved the aroma complexity of wines by increasing esters and terpenes when inoculated with *S. cerevisiae* strains compared to inoculation with *S. cerevisiae* strains alone. NM218 resulted in a more positive effect on CS wine aroma, with higher levels of citronellol and *trans*-nerolidol. BF345 significantly enhanced the floral and fruity aromas of CH wine by producing higher concentrations of geranyl acetone, *β*-damascenone, *trans*-nerolidol, and nerol. Both NM218 and BF345 yeasts could potentially be used to improve wine aroma and overall quality, especially wine floral and fruity aromas, when used in sequential inoculation with *S. cerevisiae*.

## Introduction

Non-*Saccharomyces* (NS) yeasts are mainly found on grape skins and in vineyards and wineries, including *Meyerozyma*, *Torulaspora*, *Zygosaccharomyces*, *Debaryomyces*, *Hanseniaspora*, *Metschnikowia*, *Pichia*, *Issatchenkia*, *Candida*, *Kloeckera*, and *Saccharomycodes* (Ciani et al., 2010; Padilla et al., 2016; Han et al., 2021). Recent research has discovered several beneficial properties of NS yeasts for winemaking, including high glycerol production and the ability to produce a large number of extracellular enzymes (*β*-glucosidase, *α*-arabinosidase, *α*-rhamnosidase, esterase, pectinase, lipase, proteases, etc.) during fermentation, which can improve the wine’s body, taste, and aroma (Comitini et al., 2011; Gobbi et al., 2013). NS yeasts can produce *β*-glucosidase, which can enhance the aromatic complexity of wines by releasing volatile compounds (terpenes, esters, higher alcohols, acetaldehydes, organic acids, and volatile fatty acids) from glycosidic precursors (Swiegers et al., 2005; De et al., 2018; Kong et al., 2019). However, NS yeasts are generally unable to complete alcoholic fermentation due to their poor ethanol tolerance (Binati et al., 2020). A recent trend of mixed fermentation of NS yeasts and *S. cerevisiae* has already proven to be an attractive and profitable solution in enhancing the fermentation and quality of the final product (Jussier et al., 2006; Yamaoka et al., 2014).

Aroma is an important attribute of grape and wine quality as it has a significant effect on consumer acceptance (Sáenz-Navajas et al., 2016). Several groups of volatile compounds (terpenes, aldehydes, acids, higher alcohols, esters, and other minor compounds) form the aroma complexity of wine (Dzialo et al., 2017). Among them, C_13_-norisoprenoids are known to contribute to the balsamic and violet-like aromas, higher alcohols to the herbaceous notes, and esters and terpenes to the floral and fruity aromas (Swiegers et al., 2005; Dzialo et al., 2017). A variety of NS yeasts with high *β*-glucosidase have been used in wine fermentation which contributed significantly to aromatic complexity (Zhang et al., 2021). Candida glabrata D18, with greater *β*-glucosidase activity, increased the complexity and typicality of the fruity and floral aromas in Gewürztraminer wines when used in a sequential inoculation fermentation with commercial *S. cerevisiae* (Han et al., 2021). The use of *β*-glucosidase in an aromatic grape variety (Muscat) must fermentation was capable of hydrolyzing the *β*-glucosidic bonds between aroma and glycosides which improved the aroma quality of the wine (Wanapu et al., 2012). *Hanseniaspora guilliermondii* BF1 with high *β*-glucosidase activity was also used in the fermentation of Fiano wines, either in pure or mixed fermentation with *S. cerevisiae*, which resulted in wines with higher linalool and terpene-4-ol contents and increased fruity and rose aromas (Testa et al., 2020). Additionally, Hu et al. (2016) found that *β*-glucosidase of *Hanseniaspora uvarum* had the highest activity under winemaking conditions and catalytic selectivity for aromatic glycosides of C_13_-norisoprenoids and certain terpenes, improving berry, sweet, fresh floral, and nutty aromas in the wine. Moreover, *H. uvarum* with high *β*-glucosidase activity has been broadly reported for its properties and application in winemaking (Hu et al., 2018; Zhang et al., 2021). Nonetheless, there are few reports on the application of *Meyerozyma guilliermondii* in wine fermentation.

Previously, *M. guilliermondii* NM218 and *H. uvarum* BF345 were selected from spontaneous fermentation and considered candidate strains with high *β*-glucosidase activity (data not published). However, it is still not clear whether these yeast strains could improve wine quality and aroma complexity. Therefore, in this study, *M. guilliermondii* NM218 (NM218) and *H. uvarum* BF345 (BF345) were sequentially inoculated with commercial *S. cerevisiae* in Cabernet Sauvignon and Chardonnay winemaking, and the effects on the volatile compounds and sensory characteristics of the final wines were investigated. These findings may provide support for the future application of these two indigenous strains and further provide insights into the selection of indigenous strains to enhance the quality and aroma complexity of wine.

## Materials and Methods

### Yeast Strains and Growth Conditions

Strains NM218 and BF345 were isolated from Cabernet Sauvignon must in the Ningxia wine region (China) by our laboratory personnel and identified as *Meyerozyma guilliermondii* and *Hanseniaspora uvarum* through 26S rDNA D1/D2 sequencing. These strains were stored in the China Microbial Species Conservation Center (CGMCC) as No.23154 and No.23155, respectively. *Saccharomyces cerevisiae* Vintage Red^®^ (VR) and Aroma White^®^ (AW) purchased from Enartis (Italy) were used for Cabernet Sauvignon and Chardonnay, respectively.

The strains NM218 and BF345 were cultured at 28°C in a yeast extract peptone dextrose (YPD) medium, consisting of glucose 20 g/l, peptone 20 g/l, and yeast extract 10 g/l. Wallerstein laboratory (WL) nutrient agar medium was provided by Auboxing Biotechnology (Beijing, China) and used for viable cell counts. Also, the colony morphology of the strain on WL was used to identify other yeasts (*Pichia kluyveri* and *Torulaspora delbrueckii*) in the Laboratory-Scale Fermentation.

### Evaluation of Inoculation Strategy

Yeast population monitoring of NM218 and BF345 were performed in the synthetic must according to the method of Chassagne et al. (2005). Samples were taken at 24 h intervals. The samples were diluted to 10^−4^ and 10^−5^ in gradient of sterile water and plated on WL medium, incubated at 28°C for 72 h and then colonies were counted. The colony morphology of strains NM218 (creamy white, round), BF345 (dark green, flattened), and *S. cerevisiae* (white with light green, raised in the center) on WL medium was differentiated based on the statistics. The initial concentration of each strain was 10^6^ CFU/ml. Each treatment was performed at a controlled temperature of 23°C. After determining the timing of sequential inoculation, experiments were carried out in synthetic must with sequential inoculation of NS yeasts (NM218 and BF345) and *S. cerevisiae* to evaluate the relationship between strain biomass and *β*-glucosidase activity. Strain biomass and *β*-glucosidase activity were monitored every 24 h during fermentation until the end of fermentation.

The *β*-glucosidase activity was determined based on the procedure reported by Hu et al. (2018). Briefly, fifty milliliters (50 ml) of the sample were centrifuged for 15 min at 4°C at 8,000 rpm, and the supernatant was collected for further analysis. *β*-Glucosidase activity was then determined with a chromogenic substrate, *p*-nitrophenyl-β-D-glucopyranoside (pNPG), by adding 200 μl of enzyme to 250 μl of 750 μl citric acid-phosphate buffer, pH 5.0 (activity buffer) containing *p*NPG (1 mmol/l final concentration). The reaction mixture was incubated in a water bath at 40°C for 30 min and then 1 ml of 1 mol/l Na_2_CO_3_ was added to stop the reaction. The liberated *p*-nitrophenol (*p*NP) in this mixture was measured spectrophotometrically at 400 nm. The amount of enzyme that releases 1 μmol p-nitrophenol/min at 40°C under the specified enzyme assay conditions was considered 1 unit (U) of enzyme activity (Hu et al., 2018).

### Laboratory-Scale Fermentation of Wines

In CS winemaking, the grape was crushed and filled into a 5 l flask with the addition of 60 mg/l sulfur dioxide (SO_2_) and 20 mg/l pectinases. Chemical parameters of the final CS must were as follows: pH = 3.84, total acidity = 6.54 g/l, and reducing sugar = 268.87 g/l. Inoculation strategies of the CS must were as follows: (1) NM218 was inoculated 48 h prior to VR addition; (2) BF345 was inoculated 24 h before the addition of VR; and (3) inoculation of VR alone as the control for CS fermented wine. The wines made under the above inoculation strategies were recorded as NM218-VR, BF345-VR, and CK-VR, respectively. In CH winemaking, the grapes were pressed directly, cold clarified, and the juice transferred to 5 l flasks. Chemical parameters of the final CH must were as follows: pH = 3.53, total acidity = 6.87 g/l, and reducing sugar = 249.79 g/l. CH inoculation strategies were subsequently carried out as follows: (1) inoculation of NM218 48 h before the addition of AW; (2) inoculation of BF345 24 h prior to AW addition; and (3) inoculation of AW alone as the control for CH. The wines made following the above inoculation strategies were denoted NM218-AW, BF345-AW, and CK-AW, respectively. Fermentation of the various inoculated CS must proceeded under a controlled temperature of 25°C with daily stirring, while that of CH must proceeded at 20°C with daily shaking. Fermentation kinetics were monitored daily using reducing sugar content (OIV-MA-AS313-01: R2015, 2015), which continued until the end of the fermentation. All final wines were stored at 15°C until all analyses were carried out. The oenological parameters including alcohol, volatile acidity, total sugar, and total acidity were determined based on the methods described by OIV-MA-AS313-01: R2015 (2015).

### Identification and Quantification of Volatile Aroma Compounds

Volatile compounds were determined using headspace solid-phase microextraction–gas chromatography–mass spectrometry (HS-SPME-GC-MS) according to the method described by Wang et al. (2017) with slight modifications. The wine sample (8 ml) was added to a 20 ml sample vial containing 2.5 g NaCl and 10 μl of 2-octanol as internal standard (820.7 mg/l concentration) and then equilibrated at 40°C for 30 min with stirring (20 rpm; Duan et al., 2018). A DVB/CAR/PDMS fiber (50/30 μm film thickness, Supelco, Bellefonte, PA, United States) coupled with a manual holder was inserted to the headspace of the vial for 30 min at the same temperature with the same agitation, followed by desorption into the GC injector for 5 min at 240°C. Gas chromatography and mass spectrometry system (TRACE 1310-ISQ, Thermo Fisher Scientific, San Jose, CA, United States) equipped with a TG-WAX capillary column (60 m × 0.25 mm × 0.5 μm film thickness, Thermo) was used for the analysis of volatile compounds, and the carrier gas was helium (purity 99.999%) at a flow rate of 1 ml/min. The temperature of the GC oven started at 40°C for 5 min, then was increased to 180°C at 3.5°C/min, and maintained for 15 min. The MS transfer line and ion source temperatures were set at 180°C and 250°C, respectively. The ionization voltage (EI) was 70 eV, and the mass range was m/z 50–350.

Model wine (11% v/v ethanol, 6 g/l tartaric acid, and pH adjusted to 3.4 with 1 mol NaOH) used for drawing standard calibration curves was made using chemical standard solutions as described by Tao et al. (2008). Volatile compounds were identified according to the retention time and mass spectra of the pure standards compared with the NIST and Wiley databases (Thermo Fisher Software). Volatile identification was conducted by interpolating the relative areas versus the area of internal standard using the standard calibration curves (Supplementary Table S1). Volatiles without the available standard calibration curves were quantified using the 2-octanol (internal standard). All experiments were assayed in triplicates.

### Odor Activity Value

The odor activity value (OAV) of each volatile aroma compound was calculated as the ratio between the concentration of that compound and its perception threshold. The sum of OAV of volatile compounds (ΣOAV) from the same aroma descriptor was used to show the compounds’ contribution to the wine’s aroma profile. Based on the main odor descriptors, volatile compounds were grouped into odor series with similar aroma descriptors, and one odor series was assigned to each component (Duarte et al., 2010; Amores-Arrocha et al., 2018). Nine odor series were used to describe the flavor of the wine including fruity, sweet, floral, fatty, grassy (green), spicy, earth and mushroom, chemical, and dried fruit. This approach allows the quantitative data from the chemical analysis to be matched with the sensory analysis results to produce a sensorial description of the wine based on objective parameters (Peinado et al., 2004).

### Sensory Evaluation

Wine sensory evaluation was carried out by 10 judges consisting of members of staff and graduate students (5 men and 5 women) from Gansu Agricultural University, with more than 2 years of wine rating experience. Thirty milliliters (30 ml) of each wine sample were randomly presented to each judge in different tasting booths at 18 ± 2°C (Ovalle et al., 2021). Unsalted crackers and water were provided for the judges to cleanse their palates between samples (Andreu-Sevilla et al., 2013). Sensory attributes, including appearance (color), aroma (floral, fruity, aroma intensity, peculiar odor, and typicality), mouthfeel (acid and sweet), and overall quality (overall score), were used in the evaluation of the wine samples (Hou et al., 2020). The wine samples were quantitatively assessed using a 9-point interval scale, where 1 indicated none, 5 indicated moderate intense, and 9 indicated extremely intense.

### Data and Statistical Analysis

SPSS 19.0 software (SPSS, Inc., Chicago, IL, United States) was employed for data analysis using one-way ANOVA and principal component analysis (PCA). The significant differences were determined by the means of Duncan tests at *p* < 0.05. Excel 2016 (Microsoft, Redmond, WA, United States) and Origin 2018 (OriginLab, Inc., Northampton, MA, United States) were used to draw the figures.

## Results

### Yeast Population Dynamics and *β*-Glucosidase Activity in Synthetic Must

**Figure 1:**
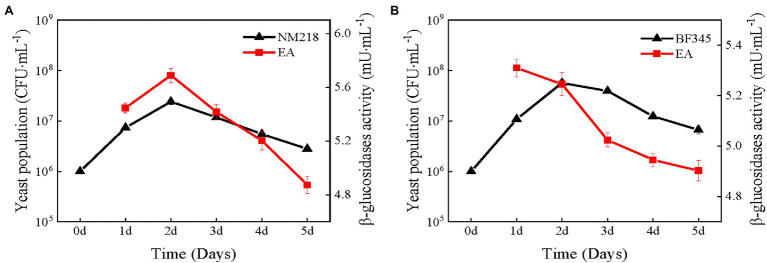
Yeast population dynamics and *β*-glucosidase activity during synthetic must fermentation. **(A)** pure fermentation of *M. guilliermondii* NM218. **(B)** pure fermentation of *H. uvarum* BF345. The black line in the graph indicates the NS yeasts population and the red line indicates the *β*-glucosidase activity (EA).

**Figure 2:**
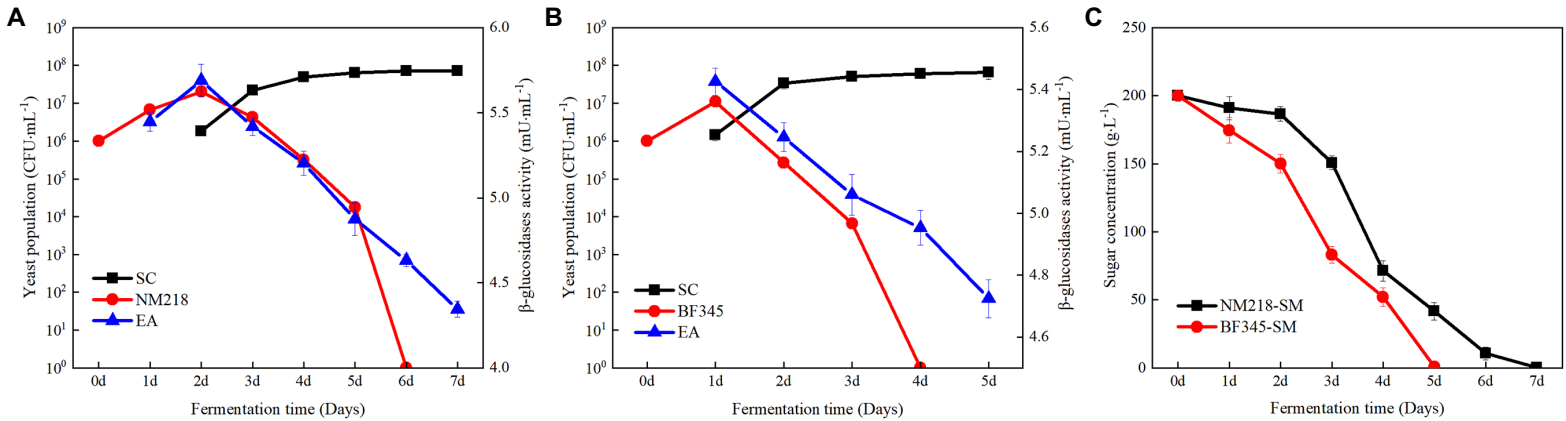
Yeast population dynamics, *β*-glucosidase activity, and sugar concentration during sequential fermentations in synthetic must. **(A)** *M. guilliermondii* NM218 and *S. cerevisiae* (VR) inoculated sequentially at 48 h intervals. **(B)** *H. uvarum* BF345 and *S. cerevisiae* (VR) sequentially inoculated at 24 h intervals. **(C)** indicates the sugar content. SC represents strains *S. cerevisiae*. NM218 and BF345 represent strains *M. guilliermondii* NM218 and *H. uvarum* BF345, respectively. The blue line indicates the *β*-glucosidase activity (EA).

The populations of strains NM218 and BFF45 were initially 10^6^ CFU/ml in synthetic must (Figure 1). The growth trends of strains NM218 and BF345 were similar. Both yeasts reached the maximum population at day 2 and then decreased as fermentation progressed; however, the peak population of NM218 (2.41 × 10^7^ CFU/ml) was lower than that of BF345 (5.69 × 10^7^ CFU/ml) during fermentation. Concurrently, the change in *β*-glucosidase activity was monitored during synthetic must fermentation. The *β*-glucosidase activity of NM218 had a similar trend to its population, reaching a maximum on day 2 (5.69 mU/ml). However, in fermentations involving BF345, *β*-glucosidase activity reached its highest level (5.31 mU/ml) on day 1 and declined thereafter as the fermentation progressed. Based on the changes in strain population and enzyme activity, the methods of sequential inoculation with *S. cerevisiae* at 48 h intervals for NM218 and 24 h intervals for BF345 were selected for winemaking. As shown in Figure 2, it was found that *β*-glucosidase activity and strain biomass decreased rapidly when NS yeasts were sequentially inoculated with *S. cerevisiae* in the synthetic must. This indicates that the biomass of NS strains may be closely related to *β*-glucosidase activity.

### Yeast Population Dynamics and Physicochemical Analysis of Wines

**Figure 3:**
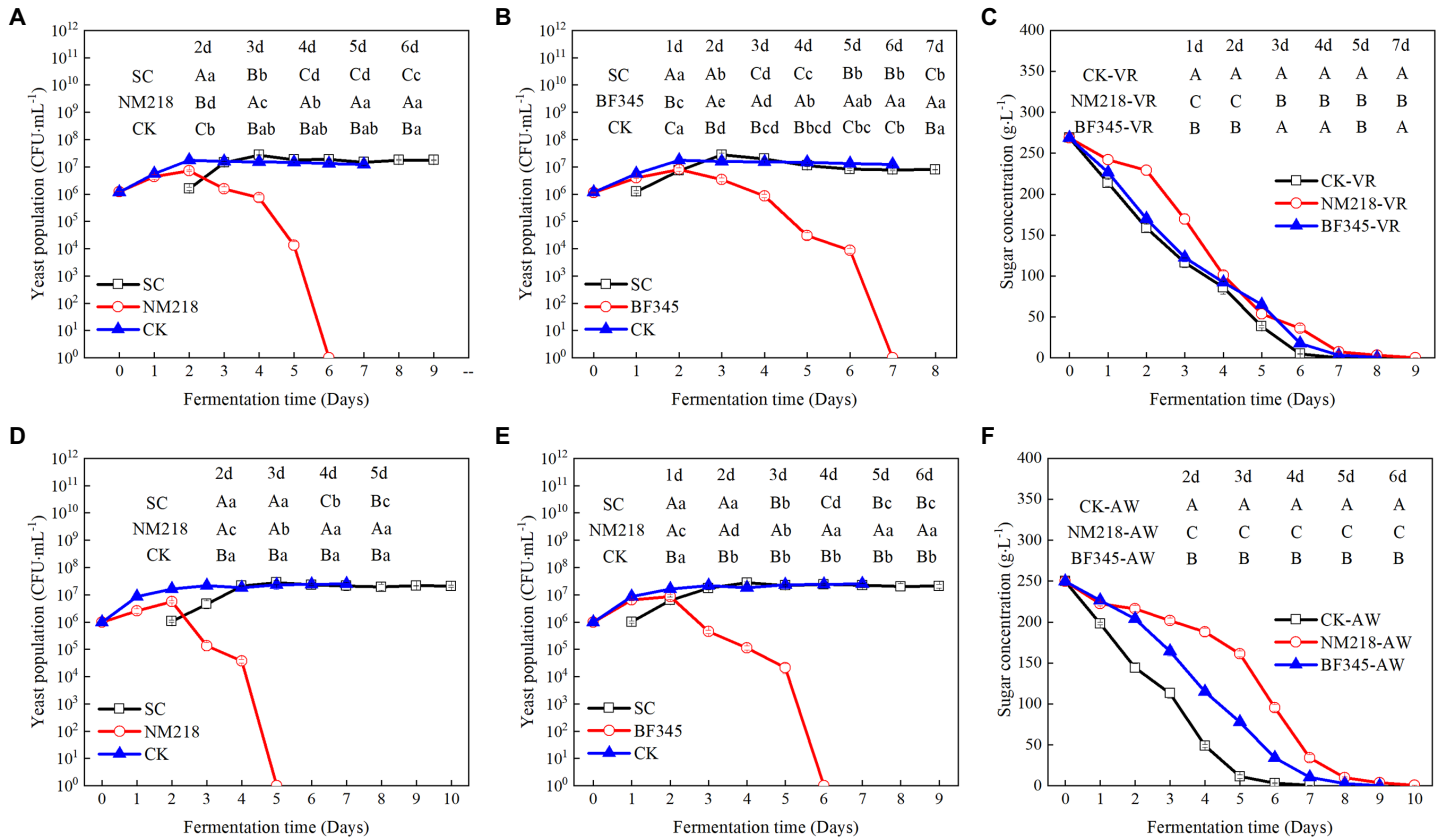
Yeast population dynamics and sugar concentration during sequential fermentations in Cabernet Sauvignon and Chardonnay must. **(A,B)** yeast population dynamics during Cabernet Sauvignon must fermentation and **(D,E)** yeast population dynamics during for Chardonnay must fermentation. **(C)** change in the sugar content during Cabernet Sauvignon must fermentation. **(F)** change in the sugar content during Chardonnay must fermentation. CK represents strains *S. cerevisiae* (control). NM218 and BF345 represent strains *M. guilliermondii* NM218 and *H. uvarum* BF345, respectively. SC represents the strains *S. cerevisiae* in sequential inoculation. CK-VR; pure culture of *S. cerevisiae* (VR) used as a control in Cabernet Sauvignon wines. NM218-VR; sequential inoculation of *M. guilliermondii* NM218 and VR at 48 h in Cabernet Sauvignon wines. BF345-VR; sequential inoculation of *H. uvarum* BF345 and VR at 24 h in Cabernet Sauvignon wines. CK-AW; pure culture of *S. cerevisiae* (AW) used as a control in Chardonnay wines. NM218-AW; sequential inoculation of *M. guilliermondii* NM218 and AW at 48 h in Chardonnay wines. BF345-AW; sequential inoculation of *H. uvarum* BF345 and AW at 24 h in Chardonnay wines. Different letters (a,b,c,d,e) in the same row for each fermentation time indicate significant differences (Duncan tests; *p* < 0.05). Different letters (A,B,C) in the same column for different yeast populations indicate significant differences (Duncan tests; *p* < 0.05).

Figure 3 shows the changes in yeast strain biomass and sugar content during the sequential inoculation fermentations of CS and CH wines. The populations of strains NM218 and BFF45 were initially 1 × 10^6^ CFU/ml in CS and CH must, respectively. The biomass of strain NM218 reached a maximum of 7.23 × 10^6^ CFU/ml (Figure 3A) and 5.56 × 10^6^ CFU/ml (Figure 3D) on the day 2 after inoculation for both strains in CS and CH must, respectively. The strain BF345 reached a maximum biomass of 8.11 × 10^6^ CFU/ml (Figure 3B) on day 1 in CS, while its biomass in CH reached a maximum biomass of 8.57 × 10^6^ CFU/ml (Figure 3E) on day 2. The population of NM218 was significantly affected after sequential inoculation with *S. cerevisiae*, resulting in a rapid decline in the yeast population and undetectable cell activity in the later stages of fermentation. The population of strain NM218 was significantly lower than *S. cerevisiae* strains from day 3 to day 5 of CS fermentation and day 4 of CH fermentation. Similarly, the population of strain BF345 on day 3 of CS and CH must fermentation was significantly lower than the populations of *S. cerevisiae* strains, showing a decreasing trend and an undetectable cell activity in the later stages of fermentation. Furthermore, Supplementary Figure S1 showed that after 24 h of inoculation with strains NM218 and BF345, the other yeast strains including *S. cerevisiae*, *Pichia kluyveri*, and *Torulaspora delbrueckii* were observed on the WL medium; however, they were at low concentrations (<10^4^ CFU/ml) and not observed (except for *S. cerevisiae*) in the middle and later stages of fermentation. As shown in Figures 3C,F, the mixed fermentations showed slower fermentation kinetics than the respective pure *S. cerevisiae* fermentations. In particular, the fermentation kinetics of strain NM218 mixed with *S. cerevisiae* was significantly lower than the other treatments in both CS and CH wines. However, all fermentations (pure and mixed) were completed within 10 days.

Table 1 shows the changes in physicochemical indicators of CS and CH wines at the end of alcoholic fermentation when NM218 and BF345 were sequentially inoculated with *S. cerevisiae*. The residual sugar content in the wine ranged from 2.07 g/l to 3.94 g/l indicating that fermentation was completed in all tests. In NM218-VR and BF345-VR fermented CS wines, the total acid contents were significantly lower than that of the control. In addition, the volatile acidity content of the test ranged from 0.34 g/l to 0.53 g/l, with small differences between the treatments, indicating that the fermentation process of ethanol proceeded normally. In NM218-AW fermented CH wine, the alcohol content was significantly lower than that of the other fermented CH wines. There were no clear differences between the physicochemical indicators of the sequentially fermented CH wines compared with the control, except for residual sugar and alcohol (Table 1).

**Table 1:** Physicochemical indicators of Cabernet Sauvignon and Chardonnay wine samples produced under different inoculation methods.

**Treatment**	**Residual sugar (g/L)**	**Total acid (g/L)**	**Volatile acid (g/L)**	**Alcohol (%, v/v)**	**pH**
**Cabernet sauvignon**					
CK-VR	0.29 ± 0.15^a^	6.10 ± 0.06^c^	0.53 ± 0.10^b^	15.70 ± 0.26^b^	3.61 ± 0.03^a^
NM218-VR	0.31 ± 0.17^a^	5.12 ± 0.11^a^	0.42 ± 0.09^a^	15.77 ± 0.45^b^	3.60 ± 0.03^a^
BF345-VR	0.41 ± 0.17^b^	5.58 ± 0.20^b^	0.34 ± 0.03^a^	14.97 ± 0.31^a^	3.58 ± 0.02^a^
**Chardonnay**					
CK-AW	0.43 ± 0.61^ab^	5.72 ± 0.45^a^	0.32 ± 0.01^a^	14.60 ± 0.26^b^	3.36 ± 0.01^a^
NM218-AW	0.51 ± 0.04^b^	5.93 ± 0.34^a^	0.30 ± 0.05^a^	14.13 ± 0.32^a^	3.31 ± 0.01^a^
BF345-AW	0.37 ± 0.18^a^	5.83 ± 0.34^a^	0.30 ± 0.01^a^	14.63 ± 0.15^b^	3.33 ± 0.01^a^

*Data are means ± standard deviation of three replicates. Data with different superscript letters (a,b,c) within each column are significantly different (Duncan tests; P < 0.05). CK-VR; pure culture of *S. cerevisiae* (VR) used as a control in Cabernet Sauvignon wines, NM218-VR; sequential inoculation of *M. guilliermondii* NM218 and VR at 48 h in Cabernet Sauvignon wines, BF345-VR; sequential inoculation of *H. uvarum* BF345 and VR at 24 h in Cabernet Sauvignon wines, CK-AW; pure culture of *S. cerevisiae* (AW) used as a control in Chardonnay wines, NM218-AW; sequential inoculation of *M. guilliermondii* NM218 and AW at 48 h in Chardonnay wines, and BF345-AW; sequential inoculation of *H. uvarum* BF345 and AW at 24 h in Chardonnay wines*.

### Effects of Sequential Fermentations on the Volatile Aroma Compounds in Wines

Volatile compounds in the wines were detected by SPME-GC–MS analysis (Table 2). Forty-six compounds were detected in CS wines, including 14 higher alcohols, 14 esters, 5 fatty acids, 7 terpenoids, 4 aldehydes and ketones, and 2 other compounds. Fifty-two compounds were detected in CH wines, including 12 higher alcohols, 19 esters, 6 fatty acids, 7 terpenes, 6 aldehydes and ketones, and 2 other compounds.

**Table 2:** Volatile compounds in Cabernet Sauvignon and Chardonnay wines.

Number	Aroma compound	Compound concentration/(mg/L)						Threshold/(mg/L)	Odor description	Odorant series
		Cabernet Sauvignon			Chardonnay		
		CK-VR	NM218-VR	BF345-VR	CK-AW	NM218-AW	BF345-AW
**Higher Alcohols**										
A1	1-Octanol	0.030 ± 0.002 b	0.032 ± 0.001 b	0.021 ± 0.001 a	0.009 ± 0.000 a	0.010 ± 0.001 a	0.010 ± 0.001 a	0.04	Jasmine, lemon scent	Floral
A2	1-Pentanol	9.892 ± 0.452 b	10.092 ± 0.336b	7.415 ± 0.222 a	5.357 ± 0.202 a	6.226 ± 1.283 b	5.717 ± 0.181 ab	64	Spicy, bready	Fatty
A3	1-Hexanol	0.178 ± 0.002 a	0.216 ± 0.006 b	0.227 ± 0.009 b	0.094 ± 0.002 a	0.093 ± 0.009 a	0.091 ± 0.005 a	8	grass	Grassy
A4	1-Decanol	0.021 ± 0.005 b	NA	0.016 ± 0.004 a	0.008 ± 0.003 a	0.007 ± 0.002 a	0.006 ± 0.002 a	0.4	Orange blossom	Floral
A5	1-Heptanol	0.065 ± 0.004 a	NA	0.064 ± 0.003 a	0.104 ± 0.003 c	0.087 ± 0.008 b	0.077 ± 0.004 a	0.2	Oily scent	Fatty
A6	1-Butanol	0.009 ± 0.005 a	0.014 ± 0.001 b	0.006 ± 0.001 a	NA	NA	NA	150	Medicinal, mellow aroma	Chemical
A7	Isobutanol	0.183 ± 0.005 a	0.181 ± 0.016 a	0.177 ± 0.016 a	0.042 ± 0.001 a	0.041 ± 0.005 a	0.040 ± 0.003 a	40	Solvent, Raw Green	Grassy
A8	2-Phenylethanol	6.319 ± 0.157 a	9.506 ± 0.660 b	9.443 ± 0.332 b	3.172 ± 0.298 a	3.690 ± 0.358 b	3.877 ± 0.117 b	1.4	Rose, honey	Floral
A9	Benzyl alcohol	0.026 ± 0.004 b	0.023 ± 0.002 b	0.017 ± 0.000 a	NA	NA	NA	200	Toasted, fruity	Fruity
A10	4-methyl-1-pentanol	0.013 ± 0.001 b	0.009 ± 0.000 a	0.007 ± 0.000 a	0.005 ± 0.000 a	0.005 ± 0.002 a	0.005 ± 0.000 a	5	Almond, Toasted	Dry fruity
A11	1-Nonanol	NA	NA	0.087 ± 0.005	0.035 ± 0.006 b	0.020 ± 0.003 a	0.031 ± 0.009 b	0.6	Fruity, Rosy	Fruity
A12	(Z)-6-nonen-1-ol	NA	NA	0.009 ± 0.003	NA	NA	NA			
A13	2-ethylhexanol	0.002 ± 0.000 a	0.003 ± 0.001 a	0.003 ± 0.001 a	0.004 ± 0.000 a	0.009 ± 0.006 b	0.007 ± 0.001 a			
A14	3-methyl-1-pentanol	NA	NA	NA	0.014 ± 0.000 a	0.014 ± 0.002 a	0.014 ± 0.001 a	1	Soil, Mushroom	Mushroom
A15	2,3-Butanediol	0.052 ± 0.017 b	0.053 ± 0.024 b	0.024 ± 0.005 a	0.007 ± 0.001 a	0.010 ± 0.001 a	0.011 ± 0.004 a	120	Butter, Cheese	Fatty
	**Total**	**16.790 ± 0.394**	**20.129 ± 0.874**	**17.516 ± 0.215**	**8.851 ± 0.076**	**10.212 ± 0.509**	**9.886 ± 1.618**			
**Esters**										
B1	Isoamyl acetate	2.000 ± 0.052 b	2.712 ± 0.107 c	1.243 ± 0.041 a	6.020 ± 0.189 a	7.110 ± 0.042 b	7.080 ± 0.150 b	0.03	Sweet and fruity, banana	Fruity
B2	Isobutyl acetate	0.005 ± 0.001 a	0.005 ± 0.002 a	0.005 ± 0.000 a	0.004 ± 0.000 a	0.005 ± 0.000 a	0.004 ± 0.001 a	1.6	Banana	Fruity
B3	Ethyl acetate	1.081 ± 0.029 a	1.096 ± 0.037 a	1.334 ± 0.045 b	0.386 ± 0.191 a	0.504 ± 0.099 b	0.518 ± 0.015 b	7.5	Fruity, sweet	Fruity
B4	Phenethyl acetate	0.296 ± 0.017 b	0.296 ± 0.022 b	0.213 ± 0.006 a	0.980 ± 0.093 b	0.738 ± 0.185 a	0.952 ± 0.155 b	0.25	Rose, Jasmine	Floral
B5	Phytol acetate	NA	NA	NA	NA	0.002 ± 0.000 a	0.003 ± 0.000 a		Fruity, sweet aroma	Fruity
B6	Geranyl acetate	NA	NA	NA	NA	0.024 ± 0.002 b	0.016 ± 0.004 a			
B7	Citronellyl acetate	NA	NA	NA	0.016 ± 0.004 a	0.010 ± 0.003 a	0.023 ± 0.006 b			
B8	Hexyl acetate	NA	NA	NA	0.488 ± 0.018 a	0.474 ± 0.010 a	0.575 ± 0.035 b	1.5	Apple, cherry, pear, floral	
B9	Neryl acetate	NA	NA	NA	0.007 ± 0.004 b	0.003 ± 0.003 a	0.003 ± 0.001 a		Apple, cherry	Fruity
B10	Isoamyl caproate	0.047 ± 0.002 b	0.043 ± 0.002 b	0.022 ± 0.003 a	0.020 ± 0.002 b	0.010 ± 0.003 a	0.021 ± 0.004 b			
B11	Ethyl octanoate	5.712 ± 0.236 b	6.416 ± 0.889 c	4.142 ± 0.115 a	7.417 ± 0.567 b	5.279 ± 2.434 a	7.322 ± 0.384 b	0.005	Rose, Orange Fruity, pineapple, pear, flora	Floral
B12	Ethyl nonanoate	0.190 ± 0.018 c	0.148 ± 0.027 b	0.125 ± 0.004 a	NA	NA	NA	1.3	Fruity, Banana	Fruity
B13	Ethyl hexanoate	1.181 ± 0.017 b	1.462 ± 0.067 c	0.800 ± 0.028 a	2.182 ± 0.097 a	2.081 ± 0.108 a	2.446 ± 0.109 b	0.08	Banana, green apple, strawberry, fennel	Fruity
B14	Ethyl decanoate	2.929 ± 0.348 b	3.196 ± 0.474 c	2.063 ± 0.126 a	1.368 ± 0.172 c	0.513 ± 0.069 a	1.014 ± 0.303 b	0.2	Coconut Fruity	Fruity
B15	Ethyl heptanoate	0.031 ± 0.001 b	0.036 ± 0.003 b	0.021 ± 0.001 a	0.012 ± 0.001 b	0.007 ± 0.000 a	0.009 ± 0.001 ab	0.22	Fruity, green, strawberry, pineapple, banana	Fruity
B16	Ethyl butanoate	0.088 ± 0.034 b	0.130 ± 0.003 c	0.039 ± 0.026 a	0.112 ± 0.003 a	0.109 ± 0.025 a	0.121 ± 0.009 b	0.02	Banana, pineapple, strawberry	Fruity
B17	Diethyl succinate	0.036 ± 0.009 a	0.076 ± 0.010 b	0.037 ± 0.002 a	NA	NA	NA	200	Fruity, Melon	Fruity
B18	Ethyl *trans*-4-decenoate	0.714 ± 0.121 c	0.295 ± 0.012 b	0.188 ± 0.031 a	1.416 ± 0.264 b	1.127 ± 0.029 a	1.474 ± 1.389 b			
B19	Ethyl palmitate	NA	NA	0.011 ± 0.005	NA	0.001 ± 0.000	NA	1.5	Floral, banana, pear	Fruity
B20	Ethyl Laurate	0.487 ± 0.140 b	0.366 ± 0.086 a	0.378 ± 0.064 a	0.378 ± 0.047 c	0.055 ± 0.018 a	0.152 ± 0.179 b	1.5	Floral, fruity, creamy	Fruity
B21	Ethyl crotonate	NA	NA	NA	0.001 ± 0.000 a	0.004 ± 0.003 a	0.004 ± 0.003 a			
	**Total**	**14.800 ± 0.601**	**16.277 ± 1.615**	**10.621 ± 0.82**	**20.810 ± 2.152**	**18.056 ± 1.011**	**21.737 ± 3.311**			
**Fatty Acids**										
C1	1-hexanoic acid	0.055 ± 0.002 b	0.064 ± 0.008 b	0.035 ± 0.003 a	NA	0.213 ± 0.017 b	0.191 ± 0.006 a	0.42	Fatty, cheesy	Fatty
C2	Isobutyric acid	0.018 ± 0.002 b	0.018 ± 0.001 b	0.008 ± 0.002 a	0.003 ± 0.000 a	0.003 ± 0.001 a	0.003 ± 0.000 a	30	Sour, cheesy	Fatty
C3	Acetic acid	0.109 ± 0.013 b	NA	0.058 ± 0.007 a	0.026 ± 0.001 b	0.021 ± 0.004 ab	0.017 ± 0.001 a	200	Acetic acid flavor	Chemical
C4	Octanoic acid	0.158 ± 0.041 b	0.169 ± 0.020 c	0.130 ± 0.021 a	0.738 ± 0.099 b	0.674 ± 0.009 a	0.677 ± 0.006 a	0.5	Putrid, pungent, cheesy	Chemical
C5	Butanoic acid	0.012 ± 0.003 a	0.017 ± 0.000 a	NA	0.007 ± 0.001 a	0.008 ± 0.003 a	0.007 ± 0.000 a	0.173	Putrid, Cheese, Candy	Chemical
C6	Decanoic acid	NA	NA	NA	0.064 ± 0.006 b	0.041 ± 0.005 a	0.039 ± 0.002 a	1	Fatty	Fatty
	**Total**	**0.352 ± 0.061**	**0.268 ± 0.029**	**0.231 ± 0.033**	**0.838 ± 0.017**	**0.960 ± 0.039**	**0.934 ± 0.015**			
**Terpenes**										
D1	Isopulegol	NA	NA	0.007 ± 0.001	NA	NA	NA			
D2	Citronellol	0.040 ± 0.009 a	0.047 ± 0.006 b	0.043 ± 0.006 a	0.036 ± 0.003 b	0.034 ± 0.002 b	0.029 ± 0.001 a	0.03	Grassy, lilac, rose	Floral
D3	Geranyl acetone	0.004 ± 0.000 a	0.005 ± 0.000 a	0.009 ± 0.001 b	0.002 ± 0.001 a	0.003 ± 0.001 a	0.004 ± 0.001 b	0.06	Light sweet, rose	Floral
D4	Geraniol	NA	0.017 ± 0.008 a	0.016 ± 0.002 a	NA	NA	0.017 ± 0.004	0.02	Lemon, peach, rose	Fruity
D5	Farnesol	0.002 ± 0.012	NA	NA	NA	NA	NA	0.02	Lime, Grass, Wood, Soft Sweet	sweet
D6	Linalool	0.030 ± 0.003 a	0.028 ± 0.010 a	0.030 ± 0.010 a	0.028 ± 0.001 a	0.030 ± 0.002 a	0.031 ± 0.003 a	0.015	Rose, citrus, fruity, sweet	sweet
D7	*Trans*-Nerolidol	0.009 ± 0.014 a	0.015 ± 0.011 b	0.013 ± 0.011 b	NA	NA	0.018 ± 0.003	0.7	Apple, rose	Fruity
D8	Nerol	NA	NA	0.003 ± 0.012	NA	NA	0.003 ± 0.000	0.4	Floral, sweet woody	Floral
D9	*β*-damascenone	NA	NA	NA	0.017 ± 0.005 a	0.025 ± 0.005 b	0.031 ± 0.015 c	0.05	Bark, canned peaches, baked apples, plums	Fruity
	**Total**	**0.085 ± 0.034**	**0.112 ± 0.035**	**0.121 ± 0.042**	**0.083 ± 0.001**	**0.092 ± 0.011**	**0.133 ± 0.021**			
**Aldehydes and Ketones**										
E1	Decyl aldehyde	0.016 ± 0.002 b	NA	0.004 ± 0.001 a	0.004 ± 0.001 a	0.004 ± 0.001 a	0.004 ± 0.001 a	1	Sweet Orange, Rose	Floral
E2	2-Octanone	NA	0.003 ± 0.001 a	0.002 ± 0.000 a	0.001 ± 0.000 a	0.001 ± 0.000 a	0.001 ± 0.000 a			
E3	2-Dodecanone	0.003 ± 0.001 a	0.002 ± 0.000 a	NA	NA	NA	NA			
E4	Nonanal	0.008 ± 0.002	NA	NA	0.003 ± 0.001 a	0.003 ± 0.001 a	0.003 ± 0.000 a	0.015	Raw green, spicy	Spicy
E5	4-phenylbutyraldehyde	NA	NA	NA	0.007 ± 0.002 b	0.004 ± 0.001 a	0.005 ± 0.001 a			
E6	Methyl heptenone	0.003 ± 0.001 a	0.005 ± 0.001 a	0.002 ± 0.000 a	0.001 ± 0.000 a	NA	0.001 ± 0.000 a			
E7	2-Undecone	NA	NA	NA	0.001 ± 0.000 a	NA	0.002 ± 0.000 a			
	**Total**	**0.030 ± 0.006**	**0.010 ± 0.000**	**0.008 ± 0.001**	**0.017 ± 0.004**	**0.012 ± 0.003**	**0.016 ± 0.002**			
**others**										
F1	2,4-Di-tert-butylphenol	0.009 ± 0.007 a	0.012 ± 0.011 a	0.021 ± 0.004 b	NA	NA	NA	0.2	Carbonic acid	Chemical
F2	2,6-Di-tert-butyl p-cresol	NA	0.140 ± 0.014 a	0.138 ± 0.017 a	0.142 ± 0.004 b	0.115 ± 0.020 a	0.128 ± 0.018 a			
F3	4-ethylguaiacol	0.071 ± 0.007	NA	NA	NA	NA	0.001 ± 0.000			
	**Total**	**0.080 ± 0.014**	**0.152 ± 0.025**	**0.159 ± 0.021**	**0.142 ± 0.004**	**0.115 ± 0.020**	**0.129 ± 0.018**			

*Data are means ± standard deviation of three replicates. Different letters (a,b,c) within the same row for each compound under each grape variety indicate significant differences in each treatment (Duncan tests; *P* < 0.05). “NA” means not detected. CK-VR; pure culture of S. cerevisiae (VR) used as a control in Cabernet Sauvignon wines. NM218-VR; sequential inoculation of M. guilliermondii NM218 and VR at 48 h in Cabernet Sauvignon wines. BF345-VR; sequential inoculation of H. uvarum BF345 and VR at 24 h in Cabernet Sauvignon wines. CK-AW; pure culture of S. cerevisiae (AW) used as a control in Chardonnay wines. NM218-AW; sequential inoculation of M. guilliermondii NM218 and AW at 48 h in Chardonnay wines. BF345-AW; sequential inoculation of H. uvarum BF345 and AW at 24 h in Chardonnay wines. Bold values represent the total content of a class of substances. A denotes higher alcohols, B denotes esters, C denotes fatty acids, D denotes terpenes, E denotes aldehydes and ketones, and F denotes other compounds. The light gray background represents volatile compounds with an OAV above 1. The dark gray background represents volatile compounds with OAV above 1 and with correlation of glucosidase activity*.

Higher alcohols are produced during alcoholic fermentation as secondary metabolites of yeast and can increase the complexity of wine aromas when the alcohol content in wine is below 300 mg/l. The wines produced by NM218 had the highest total higher alcohols in both CS and CH, with 20.129 mg/l and 10.212 mg/l, respectively. Furthermore, the CS and CH wines produced by NM218 and BF345 had an increased concentration of 2-phenylethanol (associated with rose and honey aromas) and an OAV above the odor threshold, which contributed positively to aroma of the wines.

Esters, including acetates and ethyl esters, are closely related to floral and fruity aromas. Higher concentrations of isoamyl acetate, phenethyl acetate, ethyl octanoate, ethyl hexanoate, ethyl decanoate, and ethyl butanoate (which impart fruity and floral notes and OAV above the odor threshold) were detected in NM218-VR fermented CS wines than in BF345-VR fermented CS wines. Furthermore, the concentration of ethyl esters, especially, ethyl octanoate, ethyl hexanoate, ethyl decanoate, and ethyl butanoate (with OAV above their odor threshold values), increased by 12.3, 23.8, 9.1, and 47.7%, respectively, in NM218-VR fermented wines compared to the control. BF345-VR wines on the other hand had lower concentrations of acetate esters compared to control. Regarding CH wines, the concentrations of some esters associated with floral and fruity descriptors such as isoamyl acetate (OAV > 1), ethyl acetate, hexyl acetate, ethyl hexanoate (OAV > 1), and ethyl butanoate (OAV > 1) increased significantly in BF345-AW fermented CH wines, whereas in NM218-AW fermented CH wines, isoamyl acetate (OAV > 1) and ethyl acetate had the highest concentrations compared to the control wines.

For fatty acids, the total contents in NM218-VR (0.268 mg/l) and BF345-VR (0.231 mg/l) were lower than in the control wines (0.352 mg/l). In addition, the total fatty acids contents of NM218-AW (0.960 mg/l) and BF345-AW (0.934 mg/l) wines were higher than those of control wines (0.838 mg/l). It was observed that only octanoic acid had an OAV greater than the odor threshold, which may have contributed poorly to the quality of the wines from the mixed fermentations.

Terpenes are strongly correlated with *β*-glucosidase activity and can impart floral and fruity aromas to wines. The NM218-VR and BF345-VR wines had higher concentrations of most terpenes in CS wines compared to control wines, including isopulegol, citronellol, geranylacetone, geraniol, trans-nerolidol, and nerol. These volatile aromatic compounds impart intense floral and fruity aromas to wine. Citronellol not only correlated with *β*-glucosidase activity, but its OAV also exceeded the threshold. In addition, mixed cultures (NM218-AW and BF345-AW) produced higher concentrations of terpenes in CH wines than in monoculture of *S. cerevisiae* (CK-AW). Geraniol, trans-nerolidol, and nerol were newly formed in the wines from mixed fermentation (NM218-AW and BF345-AW) compared with the wines from only *S. cerevisiae* fermentation. Compared to the control wine (CK-AW), NM218-AW fermented CH wines had higher concentrations of linalool (known to impart rose, citrus, fruity, and sweet notes) and geranyl acetone (known to contribute sweet and rose notes), while the BF345-AW fermented CH wine had higher concentration of linalool. These compounds positively contributed to the rose, citrus, fruity, and sweet aroma of the wines. In addition, linalool not only correlated with *β*-glucosidase activity, but its OAV also exceeded the odor threshold.

Despite the lower content of aldehydes and ketones, they have a positive effect on the complexity of the wine. In CS and CH, the type and content of aldehydes and ketones in the wines produced by NM218 and BF345 were significantly lower compared to the control, as a result of yeast metabolism to form new aroma compounds.

**Figure 4:**
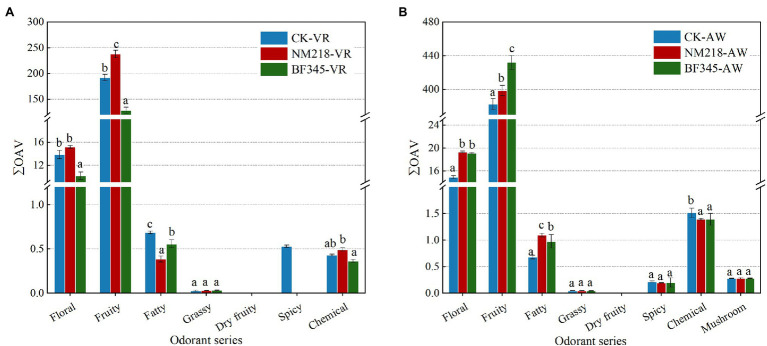
The total odor activity value (ΣOAV) of volatile aroma compounds in Cabernet Sauvignon and Chardonnay wines. **(A)** is for Cabernet Sauvignon wines and **(B)** is for Chardonnay wines. CK-VR; pure culture of *S. cerevisiae* (VR) used as a control in Cabernet Sauvignon wines. NM218-VR; sequential inoculation of *M. guilliermondii* NM218 and VR at 48 h in Cabernet Sauvignon wines. BF345-VR; sequential inoculation of *H. uvarum* BF345 and VR at 24 h in Cabernet Sauvignon wines. CK-AW; pure culture of *S. cerevisiae* (AW) used as a control in Chardonnay wines. NM218-AW; sequential inoculation of *M. guilliermondii* NM218 and AW at 48 h in Chardonnay wines. BF345-AW; sequential inoculation of *H. uvarum* BF345 and AW at 24 h in Chardonnay wines. Different letters (a,b,c) for each odor series indicate significant differences in each treatment (Duncan tests; *p* < 0.05).

Considering the ƩOAV (Figure 4), in CS, NM218-VR wines had significantly higher floral (15.20) and fruity (238.22) aromas than the BF345-VR (floral of 10.17 and fruity of 128.33) and CK-VR (13.88 for floral and 192.26 for fruity) wines. It was also observed that the NM218-VR wines had significantly lower fatty odor and higher chemical odor than the other two wines. Furthermore, the CH BF345-AW (432.13 and 19.12, respectively) and NM218-AW (398.53 and 19.34, respectively) wines showed a significant increase in fruity and floral aroma series compared to the CK-AW wines (382.71 and 14.90, respectively). It was also found that BF345-AW wines had significantly lower chemical odor than CK-AW wines but significantly higher fatty odor than CK-AW wines.

### Principal Component Analysis

**Figure 5:**
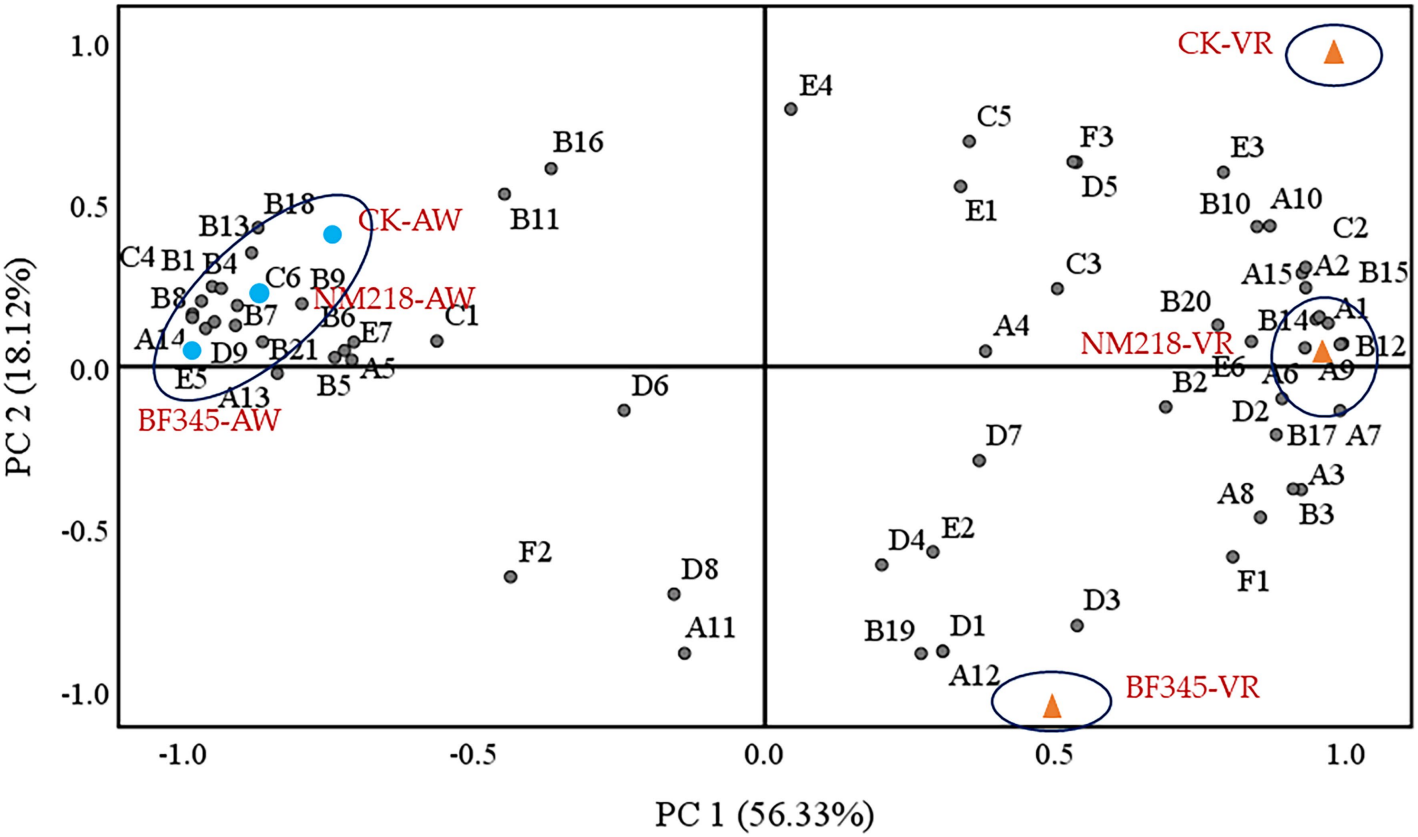
PCA of wine aroma compounds. A denotes higher alcohols, B denotes esters, C denotes fatty acids, D denotes terpenes, E denotes aldehydes and ketones, and F denotes other compounds. CK-VR; pure culture of *S. cerevisiae* (VR) used as a control in Cabernet Sauvignon wines. NM218-VR; sequential inoculation of *M. guilliermondii* NM218 and VR at 48 h in Cabernet Sauvignon wines. BF345-VR; sequential inoculation of *H. uvarum* BF345 and VR at 24 h in Cabernet Sauvignon wines. CK-AW; pure culture of *S. cerevisiae* (AW) used as a control in Chardonnay wines. NM218-AW; sequential inoculation of *M. guilliermondii* NM218 and AW at 48 h in Chardonnay wines. BF345-AW; sequential inoculation of *H. uvarum* BF345 and AW at 24 h in Chardonnay wines.

Principal component analysis (PCA) showed the correlation and separation between the aroma components and mixed fermentation methods. PC1 explained 56.33% of the total variation, while PC2 explained 18.12% (Figure 5). The NM218-AW, BF345-AW, and CK-AW fermented CH wines were categorized with 2-ethylhexanol (A13), 2-ethylhexanol (A14), isoamyl acetate (B1), phenethyl acetate (B4), geranyl acetate (B6), citronellyl acetate (B7), hexyl acetate (B8), ethyl hexanoate (B13), ethyl trans-4-decenoate (B18), ethyl crotonate (B21), octanoic acid (C4), decanoic acid (C6), and *β*-damascenone (D9) showing strong correlations. According to the PCA plot, NM218-VR, BF345-VR, and CK-VR fermented CS wines were divided into three separate groups, where NM218-VR wines were strongly correlated with 1-octanol (A1), 1-pentanol (A2), 1-butanol (A6), benzyl alcohol (A9), 2,3-butanediol (A15), ethyl nonanoate (B12), ethyl decanoate (B14), ethyl heptanoate (B15), isobutyric acid (C2) and citronellol (D2), and BF345-VR wines mainly associated with 1-nonanol (A12), ethyl palmitate (B19), isopulegol (D1), and geranyl acetone (D3), while CK-VR wines were characterized by higher accumulation of 2-dodecanone (E3).

### Sensory Evaluation of Wines

**Figure 6:**
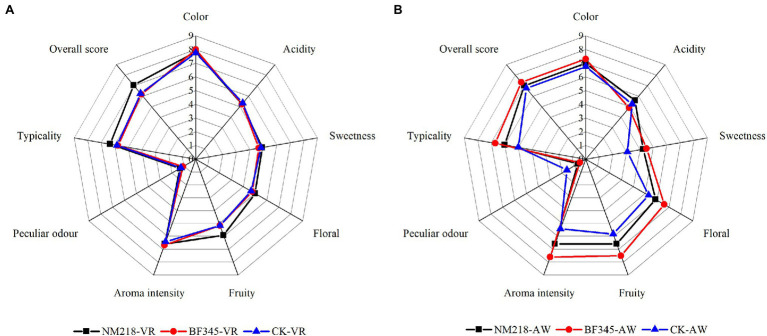
Sensory evaluation radar chart of wines. **(A)** Cabernet Sauvignon wines and **(B)** Chardonnay wines. CK-VR; pure culture of *S. cerevisiae* (VR) used as a control in Cabernet Sauvignon wines. NM218-VR; sequential inoculation of *M. guilliermondii* NM218 and VR at 48 h in Cabernet Sauvignon wines. BF345-VR; sequential inoculation of *H. uvarum* BF345 and VR at 24 h in Cabernet Sauvignon wines. CK-AW; pure culture of *S. cerevisiae* (AW) used as a control in Chardonnay wines. NM218-AW; sequential inoculation of *M. guilliermondii* NM218 and AW at 48 h in Chardonnay wines. BF345-AW; sequential inoculation of *H. uvarum* BF345 and AW at 24 h in Chardonnay wines.

To better assess the effects of the different strains on the organoleptic quality of the wines, a sensory evaluation was also performed (Figure 6). In CS wines, NM218-VR increased the fruity aroma of the wines, while BF345-VR showed no significant differences compared to the control (CK-VR). The overall score of NM218-VR wines (7.05) was higher than that of BF345-VR wines (6.17) and control wines (6.25). In the wines of CH, the wines of BF345-AW showed more fruity and floral aromas, as well as higher intensity and typicality of aromas, compared to the control wines and the wines of NM218-AW. The floral and fruity aromas, aroma intensity, and typicality of the NM218-AW wines were better than those of the control wines. Moreover, the color of BF345-AW and NM218-AW wines (golden yellow) was improved compared to the control. As for the overall score of the CH wines, the fermented CH wines of BF345-AW (7.33) were rated the best by the judges, followed by the fermented CH wines of NM218-AW (7.00).

## Discussion

The use of mixed fermentations with indigenous NS yeasts offers winemakers an opportunity to diversify regional wines (Nisiotou et al., 2018). In this study, we investigated the effect of sequential inoculation of two indigenous NS yeasts, *M. guilliermondii* NM218 and *H. uvarum* BF345, with two *S. cerevisiae* strains on the volatile composition and sensory quality of CS and CH wine based on their high *β*-glucosidase activity. In this study, consistent with Rosi et al. (1994) reported that there is a close correlation between *β*-glucosidase activity and yeast growth. Therefore, the timing of sequential inoculation intervals was determined based on the highest *β*-glucosidase activity and yeast growth. In this study, it was also found that *β*-glucosidase activity decreased rapidly when NS yeasts were sequentially inoculated with *S. cerevisiae* in the synthetic must. This could also indicate a correlation between *β*-glucosidase activity and NS yeasts biomass.

As shown in Supplementary Figure S1, other yeast strains were also observed in the WL medium, but they had much lower biomass during fermentation than the inoculated NS yeasts. Therefore, the effects of the other strains on the wine were ignored. It has been found that NS yeasts are able to improve the flavor profile of wines when they reach a biomass of 10^6^–10^7^ CFU/ml (Lema et al., 1996; Du Plessis et al., 2017). In this study, the biomass of strain NM218 at day 4 of CS and CH wine fermentation and that of strain BF345 at day 3 of CS wine fermentation and day 4 of CH wine fermentation decreased to below 10^6^ CFU/ml. Also, cell activity was undetected in strains NM218 and BF345 at the later stages of mixed fermentation. Bordet et al. (2020) reported that most NS yeasts could not be detected in the later stages of mixed fermentation. The study suggests that microbial-microbial interactions and changes in ethanol concentration are potentially important factors influencing yeast succession (Lage et al., 2014).

*Meyerozyma guilliermondii* is widely used for industrial enzyme production, metabolite synthesis, and biocontrol (Yan et al., 2020). However, there is limited information on its application in wine fermentation, especially on its effects on wine aroma and quality. The use of *H. uvarum* in wine has been extensively reported, but the high variability among different *H. uvarum* strains leads to differences in the sensory profiles of the wines produced (Tristezza et al., 2016; Pietrafesa et al., 2020). In this study, *M. guilliermondii* NM218 and *H. uvarum* BF345 were found to contribute positively to the aroma and quality of CS and CH wines. However, NM218 performed better in the CS wines, while BF345 had a more positive effect on the CH wines.

In the CS wines, NM218 significantly increased the concentrations of ethyl octanoate, ethyl hexanoate, ethyl decanoate, and ethyl butanoate (their OAV >1). These are essential for the aroma of wine and are the most important odorants for the perception of fruity and floral aromas. Amorim et al. (2016) confirmed that the concentrations of ethyl esters in cachaça produced from a mixed inoculation of *S. cerevisiae* and *Meyerozyma* sp. were higher than when pure *S. cerevisiae* was used. However, the CS wine produced with BF345 showed only a significant increase in ethyl acetate. This could be due to the fact that yeast-yeast interactions can influence their formation (Swiegers et al., 2005). Additionally, mixed fermentation of NM218 and *S. cerevisiae* significantly increased the concentration of 2-phenylethanol in CS wines, which can impart the aromas of “rose” and “honey” to the wine. Although the expected increase in 2-phenylethanol content was not correlated with *β*-glucosidase activity, this increase could be explained by the general metabolism of yeast strains (Rodrígues et al., 2010). The *β*-glucosidase activity of strains NM218 and BF345 had enhanced the terpene compounds in wines. In this study, the CS wines produced by NM218 had increased total terpenes content (0.114 mg/l) compared with the control, enhanced with floral and fruity notes. In particular, citronellol, geraniol, and farnesol were found in the highest concentrations. Xueyan et al. (2021) also reported that *M. guilliermondii* GXDK6 with high *β*-glucosidase activity increased the concentration of terpenes by releasing glycosidic conjugates, resulting in higher floral and fruity aroma intensity. The CS wines produced by BF345 had higher concentrations of geranylacetone, geraniol, trans-nerolidol, and nerol. Previous reports have claimed that *H. uvarum* strains have higher *β*-glucosidase activity, a trait that increases the content of volatile compounds such as terpenes (Martin et al., 2018), which is consistent with the results of this study.

In CH wines, the concentrations of isoamyl acetate, ethyl acetate, phenethyl acetate, ethyl hexanoate, ethyl butanoate, and ethyl trans-4-decenoate were significantly higher in wines produced from the sequential fermentation of BF345 with *S. cerevisiae* than in wines produced with *S. cerevisiae* alone, which increased the total ester content and aroma complexity of the final wines. This may be due to the specificity of *H. uvarum* for ester metabolism, as reported by Hu et al. (2018) and Ciani et al. (2006), where *H. uvarum* was able to increase the content of esters in wine by mixed fermentation with *S. cerevisiae*. In addition, the higher concentration of ethyl acetate can promote a considerably high volatile acidity, a property usually associated with *H. uvarum* metabolism (Bely et al., 2003), which negatively affects the taste of the wine. In our study, although the wines produced by BF345 had the highest amount of ethyl acetate, the threshold was not exceeded and high levels of acetic acid were not observed. However, NM218 did not increase the total ester content in CH wines. In addition, a significant increase in the concentration of 2-phenylethanol was observed in CH wines produced with strain NM218. This could be a specific metabolite of *M. guilliermondii*. Terpenes are associated with *β*-glucosidase activity and can impart intense floral and fruity aromas to wines. In the CH wines, those produced with BF345 had higher concentrations of linalool, geranylacetone, damascenone, geraniol, trans-nerolidol, and nerol, while the highest concentrations of linalool and *β*-damascenone were found in those produced with NM218. These differences in terpenes of CH wines produced by NM218 and BF345 showed that the *β*-glucosidases of the different strains are specific for precursor molecules of terpenes in grape must (Ugliano and Henschke, 2009). Notably, *β*-damascenone was detected only in CH wines. *β*-damascenone, an important olfactory compound in grapes that can directly and/or indirectly impart floral and exotic fruity notes (Guth, 1997; Pineau et al., 2007), was present at high concentrations in CH wines produced by BF345.

According to the report of Li et al. (2020), the biomass and survival time of *H. uvarum* Yun268 with high *β*-glucosidase activity were inhibited by the killer *S. cerevisiae*; however, the fruity aroma (fruity esters and ethyl acetate) also increased during co-inoculations. Therefore, in addition to the *β*-glucosidase activity, the interactions between NS yeasts and *S. cerevisiae* during sequential inoculations maybe also improve wine aroma in this study. Therefore, the role of interactions between the NM218/BF345 and *S. cerevisiae* during sequential inoculations needs to be further researched.

## Conclusion

In the present study, the sequential fermentations of non-*Saccharomyces* yeasts *M. guilliermondii* NM218 and *H. uvarum* BF345 with *S. cerevisiae* improved wine quality by enhancing the floral and fruity aromas of the wines. The analysis of the volatile compounds with GC–MS and the sensory evaluation showed that in the CS wines the *M. guilliermondii* NM218 performed better, while in the CH wines the *H. uvarum* BF345 had a more positive effect. Thus, the mixed fermentation of specific NS yeasts (with high *β*-glucosidase activity) with *S. cerevisiae* is a possible way to improve the aromatic diversity and quality of wine products.

## Data Availability Statement

The original contributions presented in the study are included in the article/Supplementary Material; further inquiries can be directed to the corresponding author.

## Author Contributions

JW, ML, and SP contributed to the experimental design. PG and LL conducted the experiments. PG, FS, and YZ analyzed the experimental data and wrote the manuscript. SP and FS contributed to the revision of the manuscript. All authors contributed to the article and approved the submitted version.

## Funding

This research was funded by the National Key Research and Development Program Projects of China (2019YFD1002500) and the Key R&D Projects in Gansu Province (20YF8NA132).

## Conflict of Interest

The authors declare that the research was conducted in the absence of any commercial or financial relationships that could be construed as a potential conflict of interest.

## Publisher’s Note

All claims expressed in this article are solely those of the authors and do not necessarily represent those of their affiliated organizations, or those of the publisher, the editors and the reviewers. Any product that may be evaluated in this article, or claim that may be made by its manufacturer, is not guaranteed or endorsed by the publisher.

## Supplementary Material

Supplementary Figure S1 Morphology of yeast colonies on WL medium during wine fermentation. **(A)** are for Cabernet Sauvignon wines and **(B)** are for Chardonnay wines. "E, M, L" represent the early, middle, and late stages of wine fermentation, respectively. **(C)** indicates the colony morphology of strains NM218 and BF345 on WL medium after 24 h of inoculation with NS yeasts in the early fermentation of Cabernet Sauvignon must. **(D)** indicates the colony morphology of strains NM218 and BF345 on WL medium after 24 h of inoculation with NS yeasts in the early fermentation of Chardonnay must. The morphology of the yeast colonies observed on WL medium were S. cerevisiae as white with light green, raised in the center; NM218 as creamy white, round; BF345 as dark green, flattened; *Torulaspora delbrueckii* as cream-colored, spherically raised, smooth; and *Pichia kluyveri* as light green with white rings, higher protrusions, smooth.

Supplementary Table S1 Qualitative and quantitative information of twenty-six chromatographically pure standards and their calibration curves, R2 values, and linear ranges.
