# Supplementary material for: Indigenous Non-Saccharomyces Yeasts With β-Glucosidase Activity in Sequential Fermentation With Saccharomyces cerevisiae: A Strategy to Improve the Volatile Composition and Sensory Characteristics of Wines
Source: Front Microbiol. 2022 May 12;13:845837. doi: 10.3389/fmicb.2022.845837 (PMC9133630; doi:10.3389/fmicb.2022.845837)
Supplement: Supplementary Figure S1 — Morphology of yeast colonies on WL medium during wine fermentation. (A) are for Cabernet Sauvignon wines and (B) are for Chardonnay wines. "E, M, L" represent the early, middle, and late stages of wine fermentation, respectively. (C) indicates the colony morphology of strains NM218 and BF345 on WL medium after 24 h of inoculation with NS yeasts in the early fermentation of Cabernet Sauvignon must. (D) indicates the colony morphology of strains NM218 and BF345 on WL medium after 24 h of inoculation with NS yeasts in the early fermentation of Chardonnay must. The morphology of the yeast colonies observed on WL medium were S. cerevisiae as white with light green, raised in the center; NM218 as creamy white, round; BF345 as dark green, flattened; Torulaspora delbrueckii as cream-colored, spherically raised, smooth; and Pichia kluyveri as light green with white rings, higher protrusions, smooth. [file Table_1.DOCX]

Supplementary Material

**Supplementary Table 1 |** Qualitative and quantitative information of twenty-six chromatographically pure standards and their calibration curves, R^2^ values, and linear ranges.

| **Compounds** | **Purity** | **CAS in Sigma** | **CAS** | **Density** | **Linear rang (mg/L)** | **Calibration curves** | **R^2^ value** |
| --- | --- | --- | --- | --- | --- | --- | --- |
| Isobutyl acetate | >99.8%(GC) | 94823-1ML-F | 110-19-0 | 0.867 g/mL at 25 °C | 0.004-0.54 | y = 3×10^8^x - 1×10^6^ | 0.9998 |
| 1-Butanol | 99.9%(GC) | 537993-100ML | 71-36-3 | 0.81 g/mL at 25 °C | 3.04-10.13 | y = 1×10^6^x - 2×10^6^ | 0.9774 |
| *cis*-3-hexen-1-ol | ≥98.0%(GC) | 91316-100MG | 928-96-1 | 0.848 g/mL at 25 °C | 1.06-3.71 | y = 1×10^7^x + 8×10^6^ | 0.9739 |
| Benzyl alcohol | 99.8%(GC) | 305197-100ML | 100-51-6 | 1.045 g/mL at 25 °C | 0.13-1.96 | y = 2×10^7^x - 7×10^5^ | 0.9900 |
| Butyl acetate | ≥97.0%(GC) | 73285-1ML | 123-86-4 | 0.88 g/mL at 25 °C | 0.06-1.65 | y = 5×10^7^x + 1×10^6^ | 0.9997 |
| Octyl acetate | 98.5%(GC) | 04622-1ML | 112-14-1 | 0.867 g/mL at 25 °C | 0.01-1.08 | y = 2×10^9^x + 4×10^7^ | 0.9989 |
| Ethyl isovalerate | 98.0%(GC) | 112283-100ML | 108-64-5 | 0.864 g/mL at 25 °C | 0.05-1.62 | y = 3×10^9^x - 5×10^8^ | 0.9278 |
| Ethyl heptanoate | 99.0%(GC) | 112364-100ML | 106-30-9 | 0.870 g/mL at 25 °C | 0.01-3.26 | y = 2×10^9^x + 1×10^8^ | 0.9907 |
| Methyl hexoate | ≥97.0%(GC) | 21599-1ML-F | 106-70-7 | 0.884 g/mL at 20 °C | 0.01-2.21 | y = 4×10^8^x + 2×10^7^ | 0.9747 |
| Isoamyl caproate | ≥97.0%(GC) | W207500-SAMPLE-K | 2198-61-0 | 0.860 g/mL at 25 °C | 0.11-5.38 | y = 1×10^9^x + 1×10^9^ | 0.9515 |
| Isoamyl octanoate | 98.0%(GC) | W208019-SAMPLE | 2035-99-6 | 0.861 g/mL at 25 °C | 0.01-2.15 | y = 1×10^9^x + 2×10^8^ | 0.9745 |
| Hexanol | ≥99.9%(GC) | 73117-1ML-F | 111-27-3 | 0.814 g/mL at 25 °C | 0.01-1.53 | y = 4×10^7^x + 2×10^6^ | 0.9982 |
| Hexanoic acid | ≥99.0%(GC) | 21529-5ML | 142-62-1 | 0.927 g/mL at 25 °C | 0.29-4.64 | y = 5×10^6^x + 1×10^6^ | 0.9938 |
| Isovaleric acid | ≥98.5%(GC) | 78651-1ML | 503-74-2 | 0.925 g/mL at 20 °C | 0.58-5.78 | y = 2×10^6^x + 1×10^6^ | 0.9589 |
| Decanoic acid | 99.5%(GC) | 21409-5G | 334-48-5 | 0.893 g/mL at 25 °C | 1.31-5.23 | y = 2×10^6^x + 2×10^6^ | 0.9995 |
| Octanoic acid | 99.5%(GC) | 21639-5ML | 124-07-2 | 0.910 g/mL at 25 °C | 0.11-3.41 | y = 5×10^6^x + 8×10^5^ | 0.9731 |
| Ethyl acetate | 99.8%(GC) | 270989-100ML | 141-78-6 | 0.902 g/mL at 25 °C | 1.13-7.89 | y = 2×10^6^x - 1×10^6^ | 0.9508 |
| Ethyl butyrate | 99.0%(GC) | E15701-500ML | 105-54-4 | 0.875 g/mL at 25 °C | 0.02-2.19 | y = 6×10^7^x + 3×10^6^ | 0.9970 |
| Ethyl decanoate | 99.0%(GC) | 148970-100ML | 110-38-3 | 0.862 g/mL at 25 °C | 0.11-4.31 | y = 3×10^9^x - 2×10^8^ | 0.9880 |
| Phenethyl alcohol | 99.0%(GC) | 77861-250ML | 60-12-8 | 1.020 g/mL at 20°C | 0.26-15.30 | y = 3×10^7^x + 2×10^7^ | 0.9893 |
| Hexyl acetate | 99.0%(GC) | 108154-25ML | 142-92-7 | 0.870 g/mL at 25 °C | 0.05-3.26 | y = 6×10^8^x - 7×10^7^ | 0.9212 |
| Phenethyl acetate | 97.0%(GC) | W285706-SAMPLE-K | 103-45-7 | 1.032 g/mL at 25 °C | 0.01-1.94 | y = 5×10^8^x + 5×10^6^ | 0.9932 |
| Citronellol | 95.0%(GC) | W230901-SAMPLE-K | 106-22-9 | 0.855 g/mL at 25 °C | 0.11-2.14 | y = 3×10^6^x + 1×10^6^ | 0.9963 |
| Damascenone | ≥90.0%(GC) | 30395-1ML | 23726-91-2 | 0.934 g/mL at 20 °C | 0.06-3.50 | y = 3×10^7^x + 2×10^6^ | 0.9994 |
| Farnesol | ≥97.0%(GC) | 43348-1ML | 4602-84-0 | 0.886 g/mL at 20 °C | 0.44-4.43 | y = 3×10^7^x + 5×10^6^ | 0.9515 |
| Benzaldehyde | ≥99.5%(GC) | 418099 | 100-52-7 | 1.045 g/mL at 20 °C | 0.01-1.31 | y = 4×10^8^x + 1×10^7^ | 0.9989 |
| 2-octanol | 99.5%(GC) | 74858-25mL | 123-96-6 | 0.819 g/mL at 20 °C | / | / | / |
