# Supplementary figures and images for: Indigenous Non-Saccharomyces Yeasts With β-Glucosidase Activity in Sequential Fermentation With Saccharomyces cerevisiae: A Strategy to Improve the Volatile Composition and Sensory Characteristics of Wines
Source: Front Microbiol. 2022 May 12;13:845837. doi: 10.3389/fmicb.2022.845837 (PMC9133630; doi:10.3389/fmicb.2022.845837)

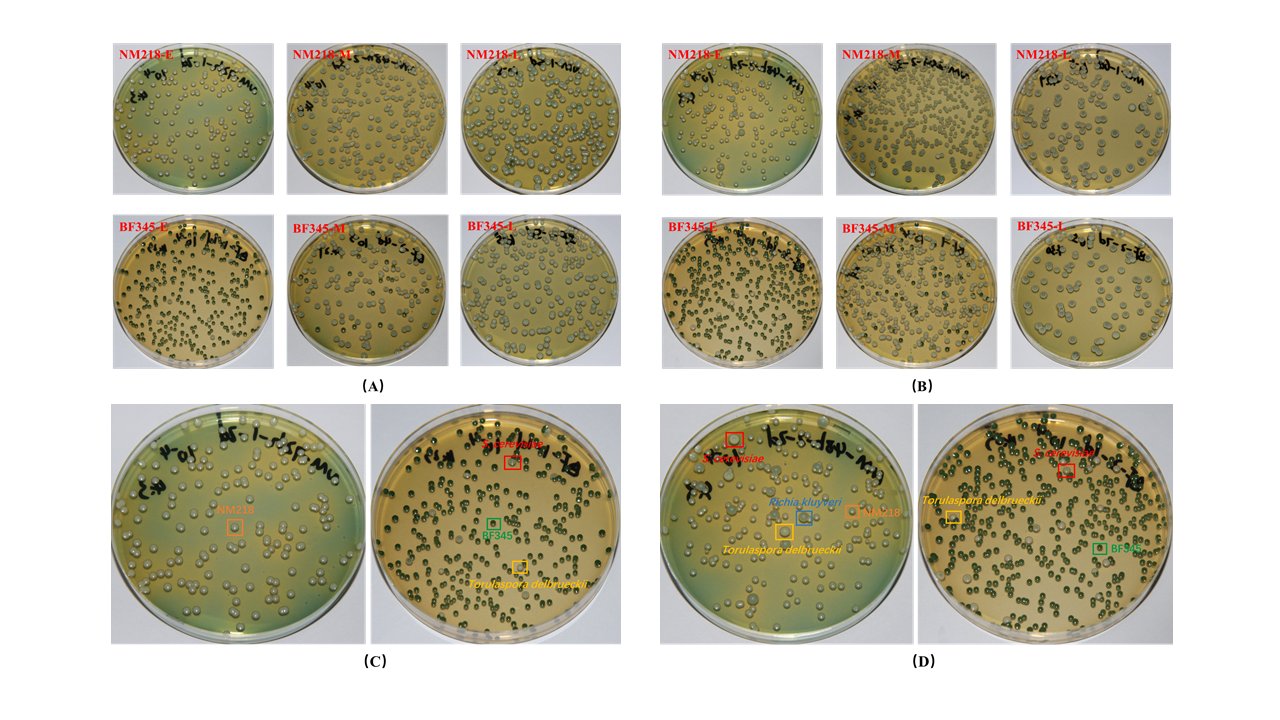

Supplement: Supplementary Table S1 — Qualitative and quantitative information of twenty-six chromatographically pure standards and their calibration curves, R2 values, and linear ranges. [file Image_1.TIF]
